# The Positive Effects of Exogenous Pancreatin on Growth Performance, Nutrient Digestion and Absorption, and Intestinal Microbiota in Piglets

**DOI:** 10.3389/fphys.2022.906522

**Published:** 2022-08-09

**Authors:** Xin Liu, Xiangyun Huang, Yang Fu, Yizhen Wang, Zeqing Lu

**Affiliations:** ^1^ National Engineering Research Center for Green Feed and Healthy Breeding, Hangzhou, China; ^2^ Key Laboratory of Molecular Animal Nutrition, Zhejiang University, Ministry of Education, Hangzhou, China; ^3^ Key Laboratory of Animal Nutrition and Feed Science (Eastern of China), Ministry of Agriculture and Rural Affairs, Hangzhou, China; ^4^ Key Laboratory of Animal Feed and Nutrition of Zhejiang Province, Hangzhou, China

**Keywords:** pancreatin, growth performance, nutrient digestion and absorption, intestinal microbiota, piglets

## Abstract

Pancreatin secretion is dramatically decreased over time after weaning, thus affecting the utilization of nutrients in piglets. Therefore, exogenous pancreatin is expected to alleviate this situation. This experiment was conducted to investigate the effects of exogenous pancreatin on the growth performance, nutrient digestion and absorption, and intestinal microbiota of piglets. One hundred eighty piglets (Duroc × Landrace × Yorkshire, 40 days) were randomly allotted to three treatments (basal diets supplemented with 0, 250, or 500 mg/kg pancreatin) with three replicate pens per treatment and 20 piglets per pen. Compared with the control diet, dietary 500 mg/kg pancreatin significantly increased (*p* < 0.05) the average daily gain (ADG) and the apparent digestibility of crude protein and crude fat of piglets. Regarding endogenous enzymes, pancrelipase activity in the pancreas, duodenal mucosa, and small intestinal digesta as well as trypsin activity in the jejunal digesta were increased in piglets fed a diet supplemented with 500 mg/kg pancreatin (*p* < 0.05). Moreover, amylopsin activity was significantly strengthened in the pancreas, duodenal mucosa, and digesta in piglets fed a diet with 500 mg/kg pancreatin (*p* < 0.05). The mRNA expression of nutrient transporters, including oligopeptide transporter-1 (PepT1), excitatory amino acid transporter-1 (EAAC1), cationic amino acid transporter-1 (CAT1), sodium glucose cotransporter-1 (SGLT1), glucose transporter-2 (GLUT2), and fatty acid transporter-4 (FATP4), in the jejunum significantly increased after dietary supplementation with 500 mg/kg pancreatin (*p* < 0.05). An increased villus height-to-crypt depth ratio of the ileum was observed in the 500 mg/kg pancreatin-treated group (*p* < 0.05). The composition of the colonic microbiota modulated by the addition of 500 mg/kg pancreatin was characterized by an increased relative abundance of *Lactobacillus* (*p* < 0.05), and the predicted functions revealed that 500 mg/kg pancreatin supplementation enhanced the functional abundance of genetic information processing in colonic microorganisms and environmental information processing. Our findings suggested that the addition of 500 mg/kg pancreatin improved the growth performance of piglets, improved intestinal structure, and modulated the colon microbiota, thereby increasing nutrient digestibility.

## 1 Introduction

Nutrients, such as carbohydrates, proteins, or lipids, are ingested and gradually digested by a series of enzymatic reactions. Eventually, all the nutrients pass through the intestinal epithelium and enter the blood circulation. Nutrient transporters expressed on the apical membrane of intestinal absorptive cells are directly exposed to an environment that changes significantly with diet; consequently, their expression is adaptively regulated by dietary substrates, including GLUT2, PepT1, and FATP4.

Pigs are easily prone to suffer weaning stress caused by their immature immune and digestive systems and sudden changes in diet and housing environment, which may be implicated in a reduction in feed intake and nutrient absorption, an increase in intestinal disorder, and insufficient secretion of endogenous enzymes, such as trypsin, pancrelipase, or amylopsin (Yin et al., 2014; Wen et al., 2020b). Mounting evidence has explored the changes in endogenous enzyme activities in the pancreas and intestinal tract of postweaning piglets. In recent decades, researchers have found that during the first week postweaning, amylopsin and pancrelipase activity decreased significantly, and trypsin activity did not change statistically (Jensen et al., 1997). Another study also reported that trypsin and amylopsin in the pancreas and small intestine digesta of 28-day weaned piglets showed a descending trend (Owsley et al., 1986). Recently, an investigation verified that the activity of trypsin, amylopsin, and pancrelipase in the pancreas and intestinal contents all decreased for a short period of time after weaning and the decrease in pancrelipase was the most remarkable (Hedemann and Jensen, 2004). Pancreatin plays a vital role in the digestion and absorption of nutrients, and the lack of pancreatin will cause food indigestion and serious nutritional deficiency, which will eventually impair the growth performance of piglets (Solomon, 1984). Therefore, it is of great importance to supplement exogenous enzyme preparations in weaned piglets.

Preparations with multiple enzyme activities could represent a convincing strategy to improve nutrient utilization in a wide range of feed ingredients (Slominski, 2011). Low-dose phytase considerably boosted the activities of sucrase and aminopeptidase in broiler feed (Al-Qahtani et al., 2021). Another study showed that carbohydrase addition helped poultry digest non-starch polysaccharides, an antinutrient factor in grain; reduced intestinal viscosity; and regulated intestinal flora to reduce the risk of infection (Raza et al., 2019). A trial showed that the application of a pepsin and trypsin mixed enzyme preparation could completely inactivate soybean lectin and increase the utilization rate of soybean (Ma and Wang, 2010). However, studies on exogenous pancreatin addition in the feed of piglets remain rare. We hypothesized that exogenous pancreatin supplementation might improve nutrient digestibility *via* improvements in digestive enzyme activity, transporter mRNA expression, and intestinal morphology and ultimately improve the growth performance of piglets. In addition, as a digestive enzyme, we wondered whether the addition of pancreatin causes changes in the colon microbiota composition in piglets. Subsequently, we performed high-throughput sequencing to determine the changes in the colon microbiota. Hence, the objective of this study was to verify this hypothesis and provide a theoretical basis for the efficient utilization of exogenous pancreatin to alleviate the insufficient secretion of endogenous pancreatin*.*

## 2 Materials and Methods

### 2.1 Ethical Approval

The approval for animal experiments was provided by the Animal Care and Use Committee of Zhejiang University (Hangzhou, China), and the study was performed in accordance with the institutional guidelines.

### 2.2 Pancreatin

Pancreatin was provided by Shanghai Ornish Biotechnology Co., Ltd., and pancreatin supplied trypsin, pancrelipase, and amylopsin, which demonstrated activity levels of 561 U/g, 4,352 U/g, and 3,061 U/g, respectively.

### 2.3 Experimental Animals, Design, and Management

One hundred eighty piglets (Duroc × Landrace × Yorkshire, 40 days) were randomly allotted to three treatments (basal diets supplemented with pancreatin at 0, 250, or 500 mg/kg) with three replicate pens per treatment and 20 piglets per pen. Their initial body weight was approximately 13 kg. The nutrient level in the basal diet (Table 1) met the requirements recommended by Hauschild et al. (2012).

**TABLE 1:** Composition and nutrient levels of the basal diet (air-dry basis, %).

Ingredient	Content	Nutrient level^a^	Content
Corn	48.3	ME (MJ/kg)	13.99
Puffed corn	15.0	CP	18.77
Soybean meal	13.0	EE	5.55
Fermented soybean meal	6.2	CF	2.46
Extruded full-fat soybean	7.2	Crude ash	3.31
Fish meal	2.5	Ca	0.73
Whey powder	3.0	Total P	0.54
Soybean oil	2.0	Lysine	1.23
CaH_2_PO_4_	0.8		
Limestone (80 mesh)	0.8		
Acidifier	0.2		
Premix^b^	1.0		

a CP, crude protein; EE, ether extract; CF, crude fiber; and ME, metabolizable energy.

b The premix supplied the following per kilogram of diet: vitamin A, 10,000 IU; vitamin D3, 2,400 IU; vitamin E, 96 mg; vitamin B1, 4.32 mg; vitamin B2, 9.6 mg; vitamin B6, 6.72 mg; vitamin B12, 43.2 μg; folic acid, 2.4 mg; niacin, 64.8 mg; D—calcium pantothenate, 14.4 mg; biotin, 0.288 mg; choline chloride, 600 mg; vitamin D, 2,000 IU; Cu, 162.4 mg; Zn, 63 mg; Fe, 63 mg; Mn, 15.68 mg; and Se, 0.3 mg.

The trial was conducted at Dalian Chengsan Animal Husbandry Co., Ltd. Feed was provided thrice daily (06:00, 11:00, and 17:00), and the remaining feed was weighed at 05:00 the next day. During the trial period, the temperature was kept at 24 ± 0.5°C, and the relative humidity was maintained at 52.15 ± 1.5%. Keeping an eye on the health status of piglets was performed. The experiment lasted 45 days.

### 2.4 Growth Performance Index and Sample Collection

At the beginning and end of the trial, all piglets were weighed individually in a limosis status, obtaining the initial and final body weight (BW) to calculate the ADG. Average daily feed intake (ADFI) was calculated by the daily feed consumption. Then, the feed conversion ratio (FCR) was calculated using the ADFI and ADG based on the following equations: ADG = (initial body weight-final body weight)/feeding period; ADFI = total feed consumption/feeding period.

At the end of the trial (d 45), four piglets were selected per pen with a BW similar to the average BW, and blood was collected *via* jugular vein puncture. Next, the blood was incubated at room temperature for 30 min and centrifuged at 1,000 ×g for 15 min. The obtained serum was stored at −20°C until further analysis. In addition, piglets fed diets with 0 and 500 mg/kg pancreatin were selected for slaughter; among them, the two closest BW piglets were chosen in each replicate. Subsequently, small intestinal sections were removed to isolate the duodenum, jejunum, and ileum. Approximately 2 cm of each sample was cut immediately and preserved in 4% formalin solution for morphological analysis. Then, the digesta and the remainder of the parts were collected in cryovial tubes, frozen in liquid nitrogen, and finally stored at −80°C until further analysis. Meanwhile, the contents of the middle of the colon were collected at −80°C for 16S rRNA sequencing.

### 2.5 Apparent Digestibility Measurement

Fecal collection lasted for 4 days at the end of the experiment. Feces were collected from every pen immediately after excretion, dried at 60°C after mixing, and ground to pass through a 40-mesh screen (to 1 mm). The fecal samples were used for the measurement of apparent digestibility (AD) of crude protein (CP) and crude fat (EE) according to McGhee and Stein (2020).

### 2.6 Intestinal Morphology Analysis

Morphological analysis of the duodenum, jejunum, and ileum was performed according to Wen et al. (2020a). Briefly, 2-cm duodenum, jejunum, and ileum segments were dehydrated and embedded in paraffin wax before transverse sections were prepared and then stained with haematoxylin–eosin and periodic acid Schiff (PAS) to visualize intestinal morphology. The measurement of villus height and crypt depth was detected with Image-Pro Plus software (Version 6.0, Media Cybernetics, United States).

### 2.7 Digestive Enzyme Activity

The protein extraction and concentration determination of the intestinal samples in this experiment were performed using a kit from Nanjing Jiancheng Institute of Bioengineering, Nanjing, China. In brief, 0.5 g of small intestine tissue samples was collected and nine times normal saline was added. The samples were ground in a homogenizer to make a 10% tissue homogenate. The samples were centrifuged at 2,000 rpm/min for 15 min, and the supernatant was collected to measure the enzyme activity. The protein concentration in the supernatants of the digesta and mucosa was similarly assayed using a BCA kit (Nanjing Jiancheng Institute of Bioengineering, Nanjing, China).Trypsin, pancrelipase, and amylopsin in duodenal, jejunal, and ileal digesta and mucosa were assayed by reagent kits according to their instructions (Nanjing Jiancheng Institute of Bioengineering, Nanjing, China). In addition, the fundamentals of the enzyme assays were as follows: Trypsin catalyzes the hydrolysis of the ester chain of the substrate ethyl arginate, resulting in an increase in absorbance at 253 nm, from which the activity of the enzyme can be calculated; the turbidity of olive oil emulsions is due to the absorption and scattering of incident light by the gel particles; the triglycerides in the gel particles are hydrolyzed by lipase to split the gel particles, thus reducing the turbidity or light scattering; the rate of reduction is related to the lipase activity; and amylase hydrolyzes starch to produce glucose, maltose, and dextrin. The amount of starch hydrolyzed can be deduced from the shade of the blue complex, and thus the activity of the amylase can be calculated by adding iodine solution to the unhydrolyzed starch when the substrate concentration is known and in excess.

### 2.8 RNA Extraction and Real-Time PCR

TRIzol reagent (Invitrogen, United States) was used for the extraction of total RNA from the jejunum, as described in our earlier protocol (Zong et al., 2019). NanoDrop 2000 (Thermo Fisher Scientific, Waltham, United States) was used to evaluate the purity and concentration of the RNA. Next, cDNA was synthesized from RNA (2 μg) using a RevertAid RT Reverse Transcription Kit (Thermo Fisher Scientific, Waltham). q-PCR was conducted with FastStart Universal SYBR Green master mix (Roche, Mannheim, Germany) *via* a StepOnePlus Real-Time PCR system (Applied Biosystems, Foster City, United States). The PCR amplification conditions were as follows: initial denaturation at 94°C for 3 min; followed by 27 cycles of denaturation at 94°C for 30 s, annealing at 55°C for 30 s, and elongation at 72°C for 40 s; and then a final extension at 72°C for 5 min. Table 2 shows gene-specific primers for q-PCR. The reference gene GAPDH was used as an internal control. Each sample was run in triplicate, and the 2^−ΔΔCT^ method was used to evaluate the relative mRNA expression of the target gene.

**TABLE 2:** Primers for real-time PCR.

Gene	Primer sequence
PepT1	F: CAG​GCT​TGC​TAC​CCA​CTG​GCA​TTT​G
	R: TGG​GAA​ACT​TCT​TAC​TCC​GAT​GCC​T
CAT1	F: ATG​GTG​TCA​GGA​TTT​GTG​AAA​GGA​T
	R:AAGCAGATCAGGAGTGAGGCGACGA
EAAC1	F: ACA​AAG​GAA​TAC​AAA​GTC​GTA​GGC​A
	R: CAG​AGC​ATT​GAA​GAA​ATC​CAC​CAG​A
SGLT1	F: GCT​GTT​CAT​CCT​GGT​GCT​GAT​TGG​C
	R: GTC​CCC​AAA​AGG​CTC​CCT​CCT​CAT​T
GLUT2	F: AAG​TTC​AGG​GGT​GCT​ATT​GGT​GCT​C
	R: GGC​ACA​GCA​GAT​AGA​CCA​AGC​AGG​A
FTP4	F: CAT​CAA​CAC​CAA​CCT​GCG​GCG​GGA​C
	R: GGA​GCA​GAA​GAG​GCT​GAG​CGA​GGG​G
GAPDH	F: AGG​GCA​CTG​TCA​AGG​CTG​AG
	R: ACG​CTG​GGA​TGA​TGT​TCT​GG

### 2.9 16S rRNA Microbiome Analysis

The CTAB or SDS method was used to extract the DNA of the colonic content. Agarose gel electrophoresis was used to detect the purity and concentration of the DNA. An appropriate amount of sample DNA was placed in a centrifuge tube, and the sample was diluted to 1 ng/μl with sterile water. Diluted genomic DNA served as a template. Based on the selection of the sequencing area, specific primers with barcodes were designed. New England Biolabs Phusion^®^ High-Fidelity PCR Master Mix with GC Buffer and high-efficiency high-fidelity enzymes for PCR were used to ensure amplification efficiency and accuracy.

A 2% agarose gel was used for electrophoresis detection of PCR products. The same amount was mixed according to the concentration of PCR products, and 2% agarose gel electrophoresis was used to detect PCR products after mixing thoroughly. The gel recovery reagent provided by the Qiagen Company was used.

A TruSeq^®^ DNA PCR-Free Sample Preparation Kit was used for library construction. The constructed library was quantified by Qubit and Q-PCR. After the library was qualified, NovaSeq6000 was used for sequencing. The sequence data were then deposited in the Sequence Read Archive under the accession number PRJNA833068.

### 2.10 Statistical Analysis

All data are presented as the mean values ± the standard error of the mean (SEM). The data were analyzed using SPSS 16.0 software. One-way ANOVA and independent-sample T tests were used for statistical analysis. The Duncan method was used for comparative analysis, and Kolmogorov–Smirnov test was used to test whether the data conformed to a normal distribution. Data regarding microbiota community were analyzed using the free online Majorbio Cloud Platform. Metagenomes were predicted from the copy number-normalized 16S rRNA data according to a previous report (Jiang et al., 2021). Differences were considered statistically significant at *p* < 0.05.

## 3 Results

### 3.1 Effects of Exogenous Pancreatin on Growth Production in Piglets

As shown in Table 3, after 40 days of feeding, compared with the control group, 500 mg/kg pancreatin supplementation significantly increased the ADG of piglets (*p* < 0.05), and the ADFI and the FCR were not significantly different (*p* > 0.05). The indices of ADG, ADFI, and FCR were not distinguished differently between the control group and the 250-mg/kg group.

**TABLE 3:** Effect of pancreatin on the growth performance of piglets.

Item	CON	250 mg/kg Pancreatin	500 mg/kg Pancreatin
Average initial body, kg	13.22 ± 0.08	13.37 ± 0.11	13.27 ± 0.15
Average final body, kg	33.08 ± 0.67	35.04 ± 0.92	35.27 ± 0.41
ADFI, g	903.63 ± 16.85	957.70 ± 32.80	974.47 ± 13.20
ADG, g	441.24 ± 15.90^b^	481.57 ± 19.70^ab^	488.94 ± 8.96^a^
FCR	2.05 ± 0.04	1.99 ± 0.01	1.99 ± 0.07

^a,b^Within a row, different superscripts mean a significant difference (*p* < 0.05).

ADFI, Average daily feed intake; ADG, average daily gain ; and FCR, feed conversion ratio.

### 3.2 Effects of Exogenous Pancreatin on Digestibility, Intestinal Digestive Enzymes, Intestinal Nutrient Translocation, and Morphology in Piglets

#### 3.2.1 Digestibility

Table 4 shows that dietary supplementation with 500 mg/kg pancreatin remarkably increased the apparent digestibility of CP and EE (*p* < 0.05), and 250 mg/kg addition made a significantly increment in the apparent digestibility of EE (*p* < 0.05).

**TABLE 4:** Effect of pancreatin on the apparent digestibility of piglets.

Item	CON	250 mg/kg Pancreatin	500 mg/kg Pancreatin
CP, %	60.01 ± 0.71^b^	61.59 ± 1.02^ab^	63.78 ± 0.77^a^
EE, %	53.76 ± 0.71^b^	58.18 ± 2.26^a^	59.32 ± 1.84^a^

^a,b^Within a row, different superscripts mean a significant difference (*p* < 0.05).

CP, crude protein; EE, ether extract.

#### 3.2.2 Intestinal Digestive Enzyme

As shown in Table 5, compared with the control group, the addition of 500 mg/kg pancreatin enhanced the pancreatin activity of the intestine and pancreas; significantly increased (*p* < 0.05) trypsin activity in the jejunal digesta and exhibited a positive trend (*p* > 0.05) on trypsin activity of the duodenal mucosa; significantly elevated (*p* < 0.05) the pancrelipase activity of pancreas, mucosa, and digesta in duodenum, and digesta in jejunum and ileum; and remarkably enhanced (*p* < 0.05) the amylopsin activity of pancreas and mucosa and digesta in duodenum. Moreover, the amylopsin activity in the mucosa and digesta of the jejunum and digesta of the ileum also improved (*p* > 0.05).

**TABLE 5:** Effect of pancreatin on trypsin, pancrelipase, and amylopsin activity in piglets.

Item	Trypsin		Pancrelipase		Amylopsin	
	CON	500 mg/kg Pancreatin	CON	500 mg/kg Pancreatin	CON	500 mg/kg Pancreatin
Pancreas, U/mg prot	3,039.26 ± 117.42	2,852.95 ± 34.62	1,969.14 ± 47.47^b^	3,079.72 ± 17.16^a^	120.26 ± 3.27^b^	168.21 ± 1.53^a^
Duodenum						
Mucosa, U/mg prot	584.42 ± 37.53	638.10 ± 38.80	5.07 ± 0.24^b^	6.41 ± 0.12^a^	0.71 ± 0.01^b^	0.72 ± 0.01^a^
Digesta, U/mg prot	6337.87 ± 226.51	5882.46 ± 54.37	252.07 ± 4.43^b^	440.35 ± 17.38^a^	0.65 ± 0.04^b^	1.02 ± 0.04^a^
Jejunum						
Mucosa, U/mg prot	509.09 ± 26.32	501.76 ± 30.03	9.32 ± 0.17	9.35 ± 0.15	0.52 ± 0.01	0.54 ± 0.01
Digesta, U/mg prot	99,26.38 ± 121.47^b^	12,653.88 ± 317.66^a^	325.56 ± 10.45^b^	335.99 ± 20.06^a^	0.35 ± 0.04	0.37 ± 0.04
Ileum						
Digesta, U/mg prot	9,363.32 ± 235.72	9,550.92 ± 22.03	276.19 ± 6.14^b^	320.74 ± 13.89^a^	0.42 ± 0.02	0.43 ± 0.01

^a,b^Within a row, different superscripts mean a significant difference, but comparison only exists in one enzyme (*p* < 0.05).

#### 3.2.3 Intestinal Nutrient Translocators

**FIGURE 1:**
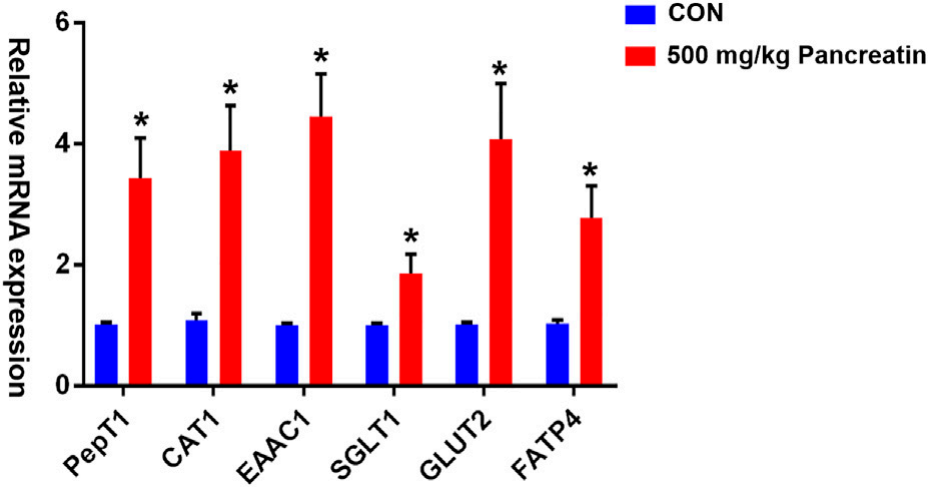
Effect of pancreatin on jejunal transporter gene expression in piglets (* represents significant differences, *p* < 0.05).

As shown in Figure 1, compared with the control group, the group supplemented with 500 mg/kg pancreatin exhibited (*p* < 0.05) a remarkable increase in the mRNA expression of PEPT1, EAAC1, CAT1, SGLT1, GLUT2, and FATP4 in the jejunum.

#### 3.2.4 Intestinal Morphology

**FIGURE 2:**
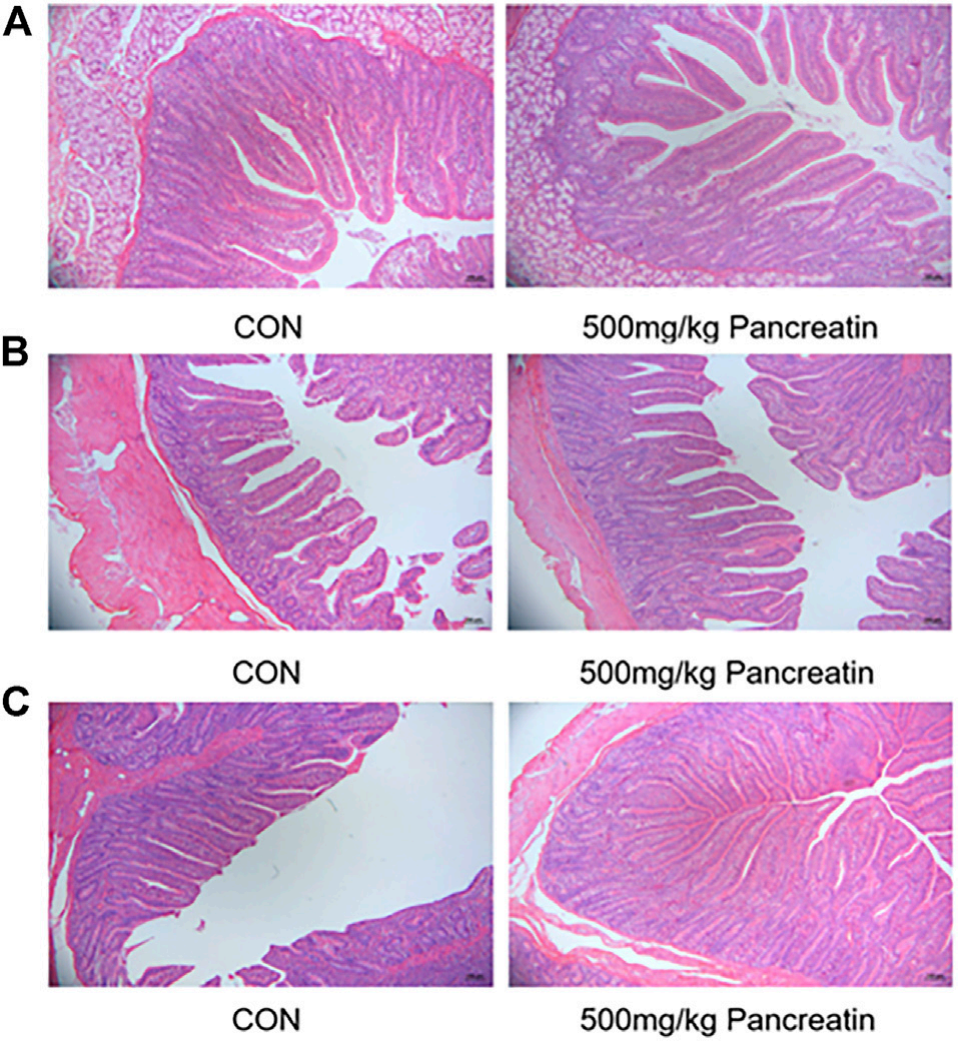
Morphology of duodenum **(A)**, jejunum **(B)**, and ileum **(C)** of piglets fed with control and 500 mg/kg pancreatin.

As shown in Table 6; Figures 2A–C, compared with the control group, the addition of 500 mg/kg pancreatin affected intestinal morphology. Specifically, an increased villus height and villus height-to-crypt depth ratio of the duodenum (*p* > 0.05) was observed. Pancreatin supplementation elevated the villus height, decreased the crypt depth, and improved the villus height-to-crypt depth ratio of the jejunum (*p* > 0.05). In addition, an increased villus height and villus height-to-crypt depth ratio of the ileum was noted (*p* < 0.05).

**TABLE 6:** Effect of pancreatin on the small intestine morphology of piglets.

Item		CON	500 mg/kg Pancreatin
Duodenum, μm	Villus height	452.44 ± 23.28	538.69 ± 35.20
	Crypt depth	359.66 ± 24.28	412.41 ± 16.51
	V:C ratio	1.28 ± 0.07	1.36 ± 0.12
Jejunum, μm	Villus height	438.53 ± 21.35	470.26 ± 28.92
	Crypt depth	251.86 ± 17.73	233.93 ± 13.52
	V:C ratio	1.79 ± 0.09	2.04 ± 0.10
Ileum, μm	Villus height	345.41 ± 17.14^b^	506.97 ± 22.78^a^
	Crypt depth	243.58 ± 20.50	253.44 ± 15.77
	V:C ratio	1.46 ± 0.12^b^	2.07 ± 0.13^a^

^a,b^Within a row, different superscripts mean a significant difference (*p* < 0.05).

V:C ratio, villus height-to-crypt depth ratio.

### 3.3 Effects of Exogenous Pancreatin on Colonic Microbiota in Piglets

#### 3.3.1 Colonic Microbial Dilution Curve of Piglets

**FIGURE 3:**
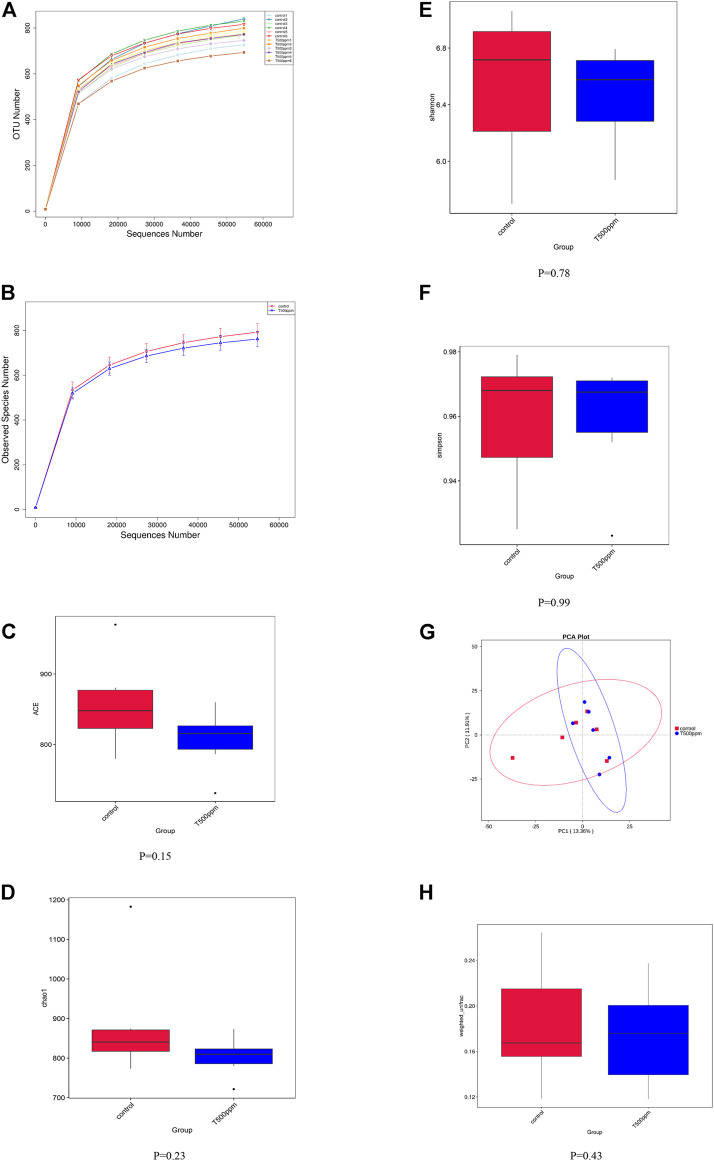
**(A,B)** Rarefaction Curve. **(C-F)** Box graph of group differences by four alpha diversity indices. **(G)** Principal Component Analysis. **(H)** Box graph of β-diversity based on Weighted Unifrac.

As shown in Figures 3A,B, the dilution curve of each sample and the dilution curve of the control group and the 500 mg/kg pancreatin group tended to be flat, indicating that the amount of sequencing data in this experiment was reasonable.

#### 3.3.2 Alpha-Diversity

Alpha diversity was used to analyze the richness and diversity of the microbial community in the sample. As shown in Figures 3C–F, no differences were detected in the alpha-diversity indices, including ACE, Chao1, Shannon, and Simpson indices, of the colonic microbiota between the control group and the 500-mg/kg pancreatin group (*p* > 0.05).

#### 3.3.3 Beta-Diversity

Beta diversity reflects the difference in microbial community composition between different samples. Principal component analysis (PCA) (Figure 3G) was performed based on the weighted UniFrac distances of the 16S rRNA sequence profiles at the OTU level. As shown in Figure 3H, the UniFrac distances suggested that both the control group and the 500 mg/kg pancreatin group showed no significant difference (*p* = 0.43).

#### 3.3.4 Differences in Microbial Flora

**FIGURE 4:**
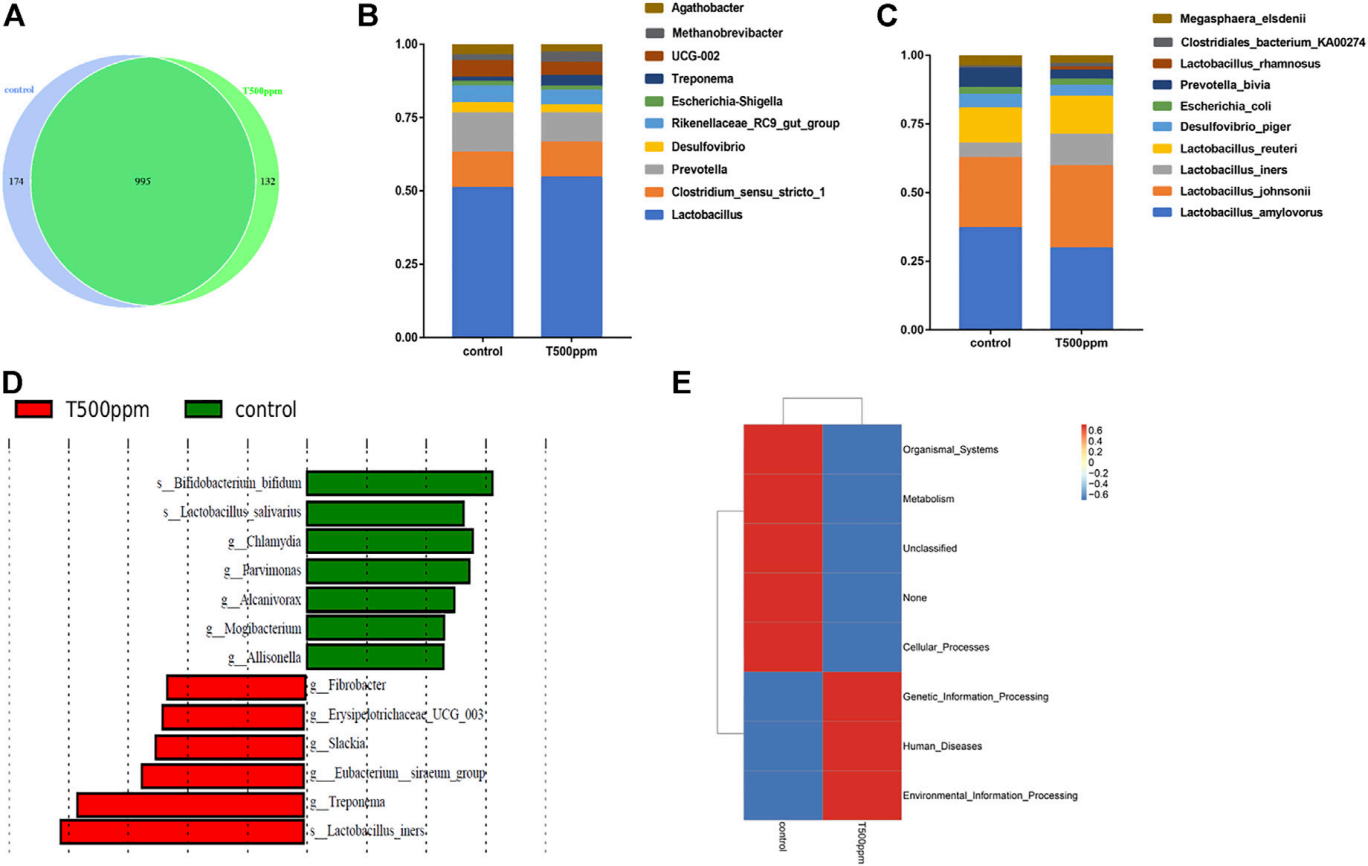
**(A)** Venn diagram of the OTU analysis of intestinal microorganisms. **(B)** TOP 10 of the genus relative abundance histogram at the genus level. **(C)** TOP 10 of the species relative abundance histogram at the species level. **(D)** LDA effect size analysis. **(E)** PICRUSt functional annotation clustering heat map.

As shown in Figure 4A, at the OTU level, 174 and 132 unique OTUs were noted in the control group and the 500 mg/kg pancreatin group, respectively. Both groups had 995 identical OTUs. As shown in Figure 4B, at the genus level, the microbiota was dominated by the genera *Lactobacillus, Clostridium sesu* stricto, and *Prevotella* followed by *Desulfovibrio*, *Rikenellaceae* RC9 gut group, *Escherichia, Shigella*, *Treponema*, UCG-002, *Methanobrevibacter*, and *Aathobacter.* Piglets fed the pancreatin diet were characterized by an increased relative abundance of *Lactobacillus*. The top 10 most abundant species are listed in Figure 4C, in which *Lactobacillus johnsonii*, *Lactobacillus iners*, and *Lactobacillus reuteri* exhibited higher abundance in the 500 mg/kg-supplemented group than the control group. The significant differentially abundant OTUs for the entire microbiota at the genus-to-species level were analyzed by LEfSe (LDA >2.0; Figure 4D). *Bifidobacteria* and *Lactobacillus salivarius* were mainly enriched in the control group, whereas *Fibrobacter, Slackia,* and *Lactobacillus iners* were mainly enriched in the pancreatin group.

#### 3.3.5 Colonic Microbial Function Prediction

To explore the effect of pancreatin on the metabolic function of bacteria, 16S rRNA sequencing results were used to predict the metabolic function of bacteria. As shown in Figure 4E, compared with the control group, the 500 mg/kg group had a higher abundance of intestinal microbes associated with genetic information processing, environmental information processing, and disease and a lower abundance of intestinal microbes associated with metabolic pathways.

## 4 Discussion

Pigs typically experience weaning stress from a sudden conversion to the environment and diet after weaning, which may lead to poor pancreatin secretion over a period of time (Marion et al., 2003). The addition of exogenous pancreatin is expected to alleviate this situation. In this study, the addition of 250 and 500 mg/kg pancreatin ameliorated the growth performance of piglets, including ADG, ADFI, and FCR, particularly ADG, in the 500 mg/kg group. Our results were consisted with those of previous research investigating the effects of the exogenous enzyme on the growth production of pigs. The addition of multi-enzyme to phytase-supplemented corn-based diet for weaned pigs could improve their growth performance and fat digestibility (Park et al., 2020). Researchers manifested that the body mass in the group of exocrine pancreatic insufficiency (EPI) pigs treated with pancreatic-like enzymes of microbial origin increased by 9% compared to the baseline (10.6 ± 2.0 kg to 11.6 ± 2.5 kg, *p* < 0.05), resulting in a reversed growth impairment in EPI pigs (Pierzynowska et al., 2018). In contrast, EPI pigs might have a similar growth status with normal pigs, which could be attributed to different ages of EPI pigs (Fedkiv et al., 2009). Increment in the digestibility of nutrients is the main reason for this phenomenon. Correspondingly, the AD of CP and EE was elevated in the 500 mg/kg supplementation group. The AD of dry matter, nitrogen, ether extract, and gross energy was increased in weaning pigs fed lipase (1.5 U/g) supplemented diets (Liu et al., 2018). Similarly, a study reported that when severe exocrine pancreatic insufficiency occurred, the extracted endogenous pancreatic enzyme from pigs could effectively improve the digestibility of fat and protein and improve the nitrogen balance *in vivo* (Van Hoozen et al., 1997). The digestibility of nutrients is closely related to digestive enzymes (de Lange et al., 2010). To fully explain the possible mechanism of the enhanced AD of nutrients, we measured the digestive enzyme activities in the pancreas and small intestinal mucosa and digesta. We observed that supplementation with 500 mg/kg pancreatin significantly increased the activity of jejunal digesta trypsin; the activities of pancreatic, duodenal mucosa and digesta, and jejunal and ileal digesta pancrelipase, and the activities of pancreatic and duodenal mucosa and digesta amylopsin, which was good for the growth performance of piglets in this study. These endogenous enzymes are paramount for the decomposition, digestion, and absorption of crude protein, lipids, and carbohydrates from large molecules to amino acids, triglycerides, and glucose in piglets (Kim et al., 2021; Long et al., 2021). Therefore, the increase in digestive enzyme activity by 500 mg/kg pancreatin could explain the amelioration of nutrient digestibility and performance in piglets. Supplementation with exogenous multienzymes enhanced the activity of amylase, lipase, and protease in all sections of the small intestine (Zhang et al., 2014). The advanced digestive enzyme activity shown in this study was possibly attributed to mixed results from ingested pancreatin and the elevated endogenous enzyme secretion caused by the increased amount of energy and nutrients available for digestion or the improved environment by the action of pancreatin (Li et al., 2004; Zhang et al., 2014). Intriguingly, the 500 mg/kg pancreatin group had lower trypsin activity in the pancreas compared with control treatment. When trypsin is expressed in large quantities, the endocrine trypsin activity is decreased, which may protect the body but does not seem to affect the activity of its exocrine trypsin (Whitcomb and Lowe, 2007). The current finding might also be because pancreatin improved the digestibility of the nutrients and supplied more substrates for these endogenous enzymes to act on, which subsequently resulted in feedback control on the secretion of the endogenous enzymes (Li et al., 2004; Fan et al., 2009). Previous studies have published that augmented nutrient digestibility is associated with augmented expression of enteral nutrient transporter genes (Heim et al., 2014; Vigors et al., 2014). Consequently, in this study, we intended to investigate whether the addition of 500 mg/kg pancreatin would amplify the gene expression of intestinal transporters. PepT1 is mainly expressed in the apical membrane of intestinal epithelial cells and is responsible for the absorption of most products of protein digestion in the body, which depends on protons to complete the efficient transport of dipeptides and tripeptides in the intestinal tract (Zuo et al., 2015; Spanier and Rohm, 2018). Correspondingly, our results revealed that the mRNA levels of jejunal PepT1 increased in the 500 mg/kg pancreatin-supplemented group compared to the control group. This finding is consistent with the results of previous studies, demonstrating that the changes in PepT1 expression resulted in a boost in CP digestibility (Habashy et al., 2017; Wani et al., 2021). PepT1 plays an imperative role in the metabolism of nitrogen throughout the body in the absorbed and postabsorbed state (Zuo et al., 2015). EAAC1 is mainly responsible for glutamate transport in the small intestine, which is Na+-dependent and closely related to the early growth and development of piglets (Kanai and Hediger, 1992). CAT1 is the main cationic transporter in the intestinal tract, and it exhibits a high affinity for lysine transport (Liao et al., 2008). In the present study, the addition of 500 mg/kg pancreatin increased the mRNA abundance of EAAC1 and CAT1 in the jejunum. Conversely, some findings determined that the expression of EAAC1 in the jejunum of weaned piglets was decreased, and the reduction of the EAAC1 gene expression was associated with an increase in glutamate transport in the jejunum of weaned piglets (Wang et al., 2017; Yu et al., 2017). This finding is in contrast to our result, which may indicate that pancreatin supplementation could explain why the mRNA expression of jejunal EAAC1 increased. In brief, the results above indicated that pancreatin addition might improve the absorption efficiency of peptides and amino acids (Zuo et al., 2015). Glucose transporters (e.g., GLUT2) and sodium-glucose cotransporters (e.g., SGLT1) are mainly involved in the transport of carbohydrates, where GLUT2 is responsible for transporting glucose across the basolateral membrane and SGLT1 is a glucose/galactose transporter expressed in the intestine (Vigors et al., 2016; Habashy et al., 2017). Our results suggested that the jejunal expression of GLUT2 and SGLT1 was increased in the 500 mg/kg pancreatin group compared with the control group. Both GLUT2 and SGLT1 expressions on the intestinal brush border layer are dependent on the luminal glucose concentration (Malunga et al., 2021). Presumably, the increased mRNA expression observed in the current study was a consequence of the addition of 500 mg/kg pancreatin. Similar experiments have revealed that supplemental carbohydrase upregulates glucose nutrient transporter gene expression (Agyekum et al., 2015; Trotta et al., 2020). FATP4 is involved in the absorption of lipids. FATP4 plays a critical role in facilitating the uptake of long-chain fatty acids into cells (Stahl, 2004). Our research confirmed that the 500 mg/kg group had higher FATP4 expression than the control group, which resulted in higher EE digestibility. Similar to our study, the antimicrobial peptide KR-32 could improve FATP4 expression through epithelial barrier recovery to increase fatty acid uptake and improve the growth performance and AD of EE in ETEC K88-challenged pigs (Liu et al., 2019)

Based on the interesting results found on the growth performance of piglets, we were further interested in whether the addition of 500 mg/kg pancreatin would influence intestinal morphology. It is well-established that intestinal development is pivotal to pig growth performance (Torres-Pitarch et al., 2017). In our study, piglets fed a basal diet with 500 mg/kg pancreatin supplementation exhibited a higher villus height and villus height-to-crypt depth ratio in the small intestine as well as an increased crypt depth in the duodenum and ileum. The increase in crypt depth indicates an increase in cell proliferation and more cells migrating to villi, and the elevated absorption area is associated with advanced villus length (Wang et al., 2019; Zhou et al., 2020). The addition of pancreatin ameliorates intestinal morphology detrimental to weaning. Exogenous enzyme supplementation is considered a good strategy to maintain and possibly improve intestinal morphology after weaning (Kim et al., 2004).

In recent years, more attention has been given to the effects of intestinal microflora on growth performance and health in pigs (Ramayo-Caldas et al., 2016; McCormack et al., 2017). Enzyme supplementation could potentially change the microbial profile and functionality in the gut by enhancing the availability of prebiotic substrates (Torres-Pitarch et al., 2017). The addition of non-starch polysaccharide enzymes reduces the fermentation substrate of the colon microbe, thus reducing the colonization rate of the intestinal microbe, which may lead to changes in the bacterial structure (Engberg et al., 2004). Data from the analysis of α-diversity and β-diversity showed that 500 mg/kg pancreatin addition did not improve microbial richness or alter the microbiota structure. In addition, dietary supplementation with increasing levels (40, 100, and 200 mg/kg) of lysozyme did not significantly affect the composition and diversity of the cecal microbiota (Xia et al., 2019), which is consistent with our results. At the genus level, there was an increased abundance of *Lactobacillus* in the colon of pancreatin-supplemented piglets. As one of the predominant genera in the piglet gut, *Lactobacillus* can protect the intestinal barrier from pathogenic bacterial damage (Servin, 2004). Moreover, lactate produced from the fermentation of *Lactobacillus* promotes the production of butyrate, which improves nutrient digestibility and intestinal morphology (De Maesschalck et al., 2015). Dietary supplementation with xylanase did not change among the abundance of *Bifidobacterium, Lactobacillus, and Escherichia coli* (Kubiś et al., 2020). Increasing β-glucanase up to 600 U/kg feed in a diet containing xylanase (1,500 EPU/kg) modulated mucosa-associated microbiota by increasing the relative abundance of beneficial bacteria and reducing potentially harmful bacteria (Duarte et al., 2021). The possible explanation is that distinct enzymes lead to distinct substrates and distinct environments in which microorganisms live, resulting in distinct microbial structures. Hence, whether the ideal enzyme at the optimal concentration can improve the intestinal microbial structure while improving growth performance and body health has not been determined. Further analysis at the species level indicated that 500 mg/kg pancreatin supplementation boosted the abundance of *Lactobacillus johnsonii, Lactobacillus iners,* and *Lactobacillus reuteri. Lactobacillus johnsonii* increased the intestinal organic acid content, improved the intestinal environment of piglets, was more conducive to the absorption of nutrients, promoted the growth of probiotics, and inhibited the proliferation of pathogenic microorganisms (Giang et al., 2010). Based on LEfSe analysis, we concluded that in the 500 mg/kg group, *Lactobacillus_iners* was increased at the species level, whereas increased abundance of *Slackia* and *Fibrobacter* was noted at the genus level. The abovementioned results showed that the addition of pancreatin could increase the abundance of beneficial bacteria in the colon of piglets, which further implied that pancreatin might regulate the microbial community structure in the colon of piglets by improving the community abundance of different beneficial bacteria and ultimately improving the performance of piglets. Gut microbes can influence host metabolism and health by secreting bioactive substances (Fiorucci and Distrutti, 2015). Therefore, we used the PICRUSt algorithm to explore the function of the microbiome. We observed that the abundance of intestinal microbes involved in genetic information processing, cell signal conduction processing, and disease increased, whereas the abundance of intestinal microbes involved in metabolic pathways decreased in the 500 mg/kg pancreatin group. We hypothesized that with the addition of pancreatin, intestinal bacteria may enhance their ability to express their own genetic information and signal transduction ability of their own cells, thus enhancing the activity of intestinal bacteria.

## 5 Conclusion

Supplementation with 500 mg/kg pancreatin improved the growth performance by modulating nutrient digestibility, which increased the digestive enzyme activity, nutrient transporter expression, and intestinal morphology and altered the colon microbiota composition in piglets. This study expands our understanding of the function of exogenous pancreatin in weaned piglets.

## Data Availability

The datasets presented in this study can be found in online repositories. The names of the repository/repositories and accession number(s) can be found below: https://www.ncbi.nlm.nih.gov/, PRJNA833068.
